# Patterns of recurrence in patients achieving pathologic complete response after neoadjuvant chemoradiotherapy for rectal cancer

**DOI:** 10.1007/s00432-017-2383-9

**Published:** 2017-04-06

**Authors:** Wen-Hua Fan, Jian Xiao, Xin An, Wu Jiang, Li-Ren Li, Yuan-Hong Gao, Gong Chen, Ling-Heng Kong, Jun-Zhong Lin, Jian-Ping Wang, Zhi-Zhong Pan, Pei-Rong Ding

**Affiliations:** 1 State Key Laboratory of Oncology in South China, Collaborative Innovation Center for Cancer Medicine, 651 Dongfeng Road East, Guangzhou, 510060 People’s Republic of China; 2 Department of Colorectal Surgery, Sun Yat-sen University Cancer Center, Guangzhou, People’s Republic of China; 3 Department of Medical Oncology, The Sixth Affiliated Hospital, Sun Yat-Sen University, Guangzhou, People’s Republic of China; 4 Department of Medical Oncology, Sun Yat-sen University Cancer Center, Guangzhou, People’s Republic of China; 5 Department of Radiation Oncology, Sun Yat-sen University Cancer Center, Guangzhou, People’s Republic of China; 6 Department of Colorectal Surgery, The Sixth Affiliated Hospital, Sun Yat-Sen University, Guangzhou, People’s Republic of China

**Keywords:** Rectal cancer, Neoadjuvant therapy, Chemoradiotherapy, Recurrence

## Abstract

**Purpose:**

The aim of this study was to characterize the patterns of recurrence in patients achieving pathologic complete response (pCR) after neoadjuvant chemoradiotherapy (CRT) for locally advanced rectal cancer.

**Methods:**

Patients with locally advanced rectal cancer treated with neoadjuvant CRT and who achieved pCR from January 2004 to December 2012 were collected. The primary outcome measurement was the patterns of recurrence.

**Results:**

Among 195 patients who achieved pCR, 18 developed recurrence. Furthermore, local recurrence occurred in 1.5% of patients (3/195), while distant metastases occurred in 7.7% of patients (15/195), which included 7 lung metastases, 1 liver metastasis, and 8 metastases in other locations.

**Conclusions:**

Our study indicated that patients achieving pCR following neoadjuvant CRT have a favorable prognosis, with distant metastases predominating in all recurrences. Among patients with distant metastases, non-liver metastases were the predominant pattern.

## Background

Preoperative chemoradiotherapy (CRT) has been used as the standard management strategy for locally advanced rectal cancer over the past decade (Sebag-Montefiore et al. 2009; Roh et al. 2009; Sauer et al. 2004), improving the outcome of locally advanced rectal cancer (Maas et al. 2010; Rodel et al. 2005; Garcia-Aguilar et al. 2003). Treatment response to neoadjuvant CRT is linked with oncologic outcomes (Park et al. 2012). Fifteen to twenty percent of patients with locally advanced rectal cancer might achieve pathologic complete response (pCR) after neoadjuvant CRT, which is associated with favorable long-term outcome (Park et al. 2012; Campos-Lobato et al. 2011; Smith et al. 2010).

Studies reporting the patterns of recurrence in patients achieving pCR after neoadjuvant CRT are very limited. In this study, we investigated the patterns of recurrence in this subgroup of patients.

## Patients and methods

We retrospectively reviewed patients with locally advanced rectal cancer treated with preoperative CRT followed by radical resection at the Sun Yat-Sen University Cancer Center and the Sixth Affiliated Hospital of Sun Yat-Sen University between January 2004 and December 2012. Patients who achieved pCR after CRT and those who developed recurrence were enrolled in the study.

Tumors were staged according to the American Joint Committee on Cancer (AJCC) Cancer Staging Manual (Edge and Compton 2010). We defined pCR as absence of viable adenocarcinoma cells in the surgical specimen (ypT0N0). Local and distant recurrence was defined as relapse within and outside the pelvis, respectively. Time to diagnosis was defined as the time between any initial abnormal findings on follow-up examination to clinical or histological confirmation of recurrence.

Pre-treatment staging, tumor location, details of neoadjuvant and adjuvant therapy, interval to surgery, type of surgery, number of lymph nodes (LNs) retrieved, site(s) of recurrence, and salvage treatment were extracted from medical records.

We excluded patients who were diagnosed with a non-skin cancer within 5 years of the rectal cancer diagnosis, did not complete neoadjuvant CRT, and who did not undergo radical rectal resection.

Pre-treatment evaluation included digital rectal examination, chest and abdominal computed tomography (CT) scans, and endoscopic ultrasound and/or magnetic resonance imaging (MRI) of the pelvis. Prior to treatment, the pathologists of each hospital confirmed that all enrolled patients had adenocarcinoma of the rectum. Patients were treated with CRT that involved radiotherapy and fluorouracil (FU)-based chemotherapy. Surgery was generally performed 4–10 weeks following completion of CRT according to total mesorectal excision (TME) principles.

Patients were followed-up every 3 months for 2 years, every 6 months for the next 3 years, and annually thereafter. Evaluations consisted of physical examination, serum carcinoembryonic antigen levels, abdominal ultrasound, and chest radiography. Liver ultrasonography was carried out every 3 months. Chest, abdominal, and pelvic CT scans were performed every year for 5 years. Other investigations were performed when clinically indicated during follow-up.

## Results

Our retrospective review determined that 771 patients with locally advanced adenocarcinoma of the rectum had undergone CRT followed by TME at the Sun Yat-Sen University Cancer Center and the Sixth Affiliated Hospital of Sun Yat-Sen University between January 2004 and December 2012. Twelve patients were excluded due to concurrent distant metastasis, concurrent malignancy, or prior history of pelvic radiotherapy. An eventual 195 patients with ypT0N0 pathology (i.e., achieved pCR) were enrolled in the present study; 18 developed recurrence. The characteristics of these 18 patients are summarized in Table 1.

**Table 1:** Details of the 18 patients with recurrence after achieving pCR

No.	Age (years)	Sex	C Staging	Tumor location (mm)	Dose (Gy)	Concurerent Chemo	CRT-surgery interval (w)	Surgery	No. of LN	Adjuvant Chemo	Location of recurrence	RFS (M)	Outcome	OS (M)
1	75	F	T3N0	50	46	Oxa + Cape	5	LAR	4	No	Lateral pelvic	32	DOD	69
2	57	M	T3N1	10	46	Oxa + Cape	5	APR	12	Yes	Lateral pelvic	7	DOD	21
3	58	M	T3Nx	40	46	Oxa + 5-FU	6	APR	4	Yes	Lateral pelvic	12	DOD	25
4	47	F	T3N1	40	46	Oxa + Cape	9	APR	9	No	Peritoneal cavity	11	DOD	17
5	56	F	T3N2	40	46	Oxa + Cape	5	APR	4	Yes	Para-aortic LN	14	DOD	33
6	54	M	T3N2	50	46	Oxa + 5-FU	4	LAR	2	No	Supraclavicular LN, bone, lung	25	DOD	39
7	70	M	T4N0	50	46	Oxa + Cape	6	LAR	0	Yes	Liver	7	DOD	15
8	32	M	T3N2	40	46	Oxa + Cape	6	APR	7	Yes	Lung	14	DOD	22
9	43	F	T3N0	70	46	Oxa + Cape	9	LAR	0	Yes	Retroperitoneal LN	19	AWD	23
10	63	M	T3N0	30	46	Oxa + Cape	10	APR	12	No	Lung	8	DOD	15
11	57	F	T3N0	30	50	Oxa + Cape	7	LAR	3	No	Lung	14	AWD	14
12	54	F	T3N1	40	50	Oxa + Cape	7	LAR	11	Yes	Bone	6	AWD	14
13	21	M	T4N0	30	46	Oxa + 5-FU	7	LAR	18	Yes	Brain	30	DOD	33
14	58	M	T3N2	45	46	Oxa + 5-FU	6	LAR	10	Yes	Retroperitoneal LN	7	AWD	9
15	38	M	T3N0	25	46	Oxa + 5-FU	8	APR	6	Yes	Lung	14	AWD	15
16	27	F	T4N0	55	46	Oxa + 5-FU	8	LAR	12	Yes	Retroperitoneal LN, lung, bone	22	DOD	24
17	54	M	T3N0	30	46	5-FU	7	LAR	9	Yes	Peritoneal cavity	19	DOD	22
18	50	F	T3N1	30	46	Oxa + 5-FU	7	LAR	14	Yes	Lung	10	AWD	49

*5-FU* 5-fluorouracil, *APR* abdominoperineal resection, *AWD* alive with disease, *Cape* capecitabine, *chemo* chemotherapy, *CRT* chemoradiotherapy, *Chemo* chemotherapy, *DOD* died of disease, *F* female, *FOLFOX6* 5-FU, leucovorin, oxaliplatin, *LAR* low anterior resection, *LN* lymph nodes, *M* male, *OS* overall survival, *Oxa* oxaliplatin, *pN (n)* number of LN retrieved, *RFS* recurrence-free survival, *RT* radiotherapy, *Tar* targeted therapy, *XELOX* capecitabine and oxaliplatin

**Fig. 1:**
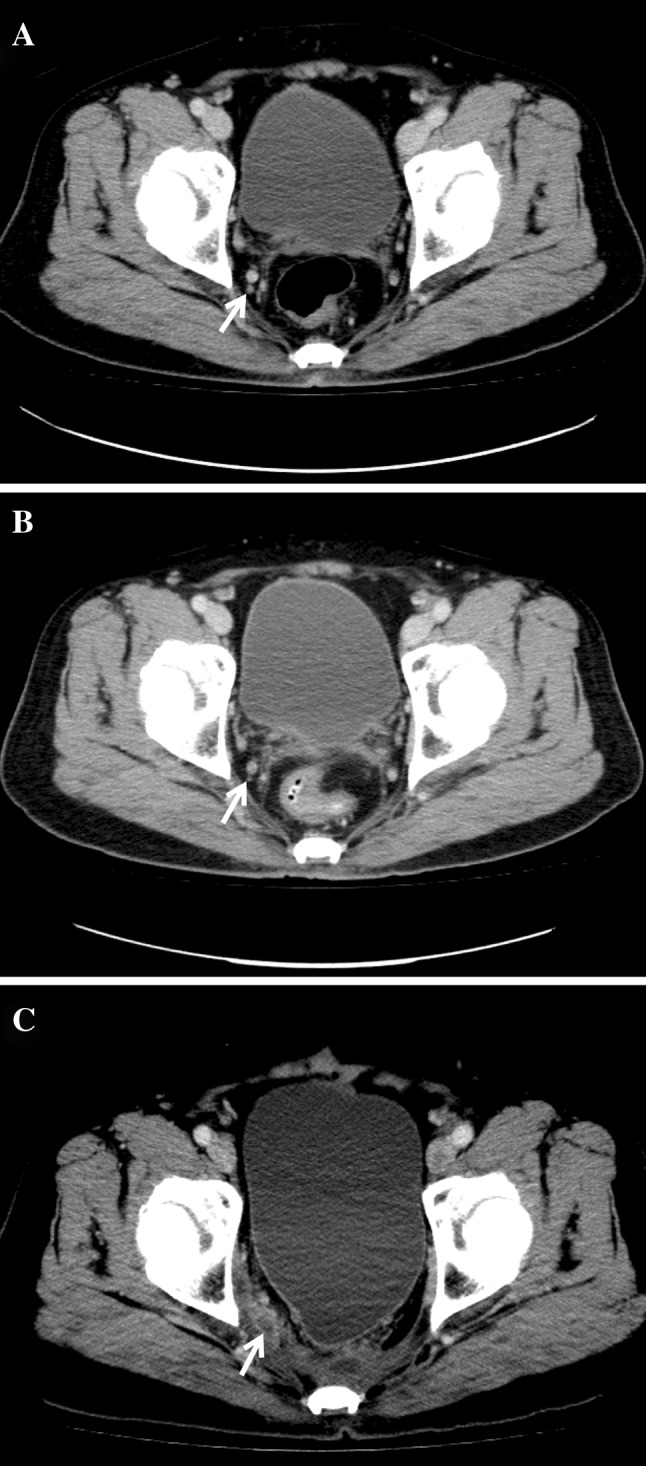
Lateral pelvic recurrence on computed tomography. **a** Pre-treatment image shows a 5-mm lymph node in the right obturator area (*white arrow*). **b** Patient underwent neoadjuvant chemoradiotherapy and the metastatic lymph node was decreased to 2 mm (*white arrow*). **c** Follow-up image at 12 months after surgery shows lateral pelvic recurrence (*white arrow*).

Three patients developed lateral pelvic sidewall recurrence. All recurrence occurred in the lower rectum (within 5 cm from the anal verge), and pre-treatment CT scan detected lateral LN metastasis (Fig. 1). After preoperative CRT, the metastatic LN was decreased to 2 mm. Standard TME was performed without lateral pelvic LN dissection (LPLD). However, there was recurrence in the lateral pelvic LN (LPLN), where recurrence occurred in 2 and 1 patient within 1 and 3 years, respectively, from the time of surgery. Only one patient underwent examination for 12 LN.

Distant metastases occurred in 7.7% of patients (15/195; median time to relapse: 14 months), including 7 lung metastases, 1 liver metastasis, and 8 metastases in other locations. In all but 2 patients, recurrence was 5 cm below the anal verge, and the median number of examined LN was 9 (range: 0–18). Four of seven lung metastases and one liver metastasis were histological confirmed. None of the patients had both local and distant recurrence.

## Discussion

Following the introduction of TME and CRT, local failure rates after curative (R0) resection of locally advanced rectal cancer have decreased dramatically (Sauer et al. 2004; Collette et al. 2007; Wibe et al. 2002). Previous studies have consistently demonstrated that patients with good response after CRT have better long-term outcomes (Garcia-Aguilar et al. 2003; Park et al. 2012).

The AJCC and International Union Against Cancer recommend retrieving a minimum 12 LN to achieve appropriate staging. However, several studies have reported lower LN yield in proctectomy specimens from patients treated with preoperative CRT (Fuente et al. 2009; Marks et al. 2010; Govindarajan et al. 2011). In the present study, we retrieved much fewer LN (median number of examined LN: 8, range: 0–18) than previous reports of patients treated with neoadjuvant CRT. As has been previously reported, the number of LN harvested varies greatly even when surgical treatment and pathological evaluation have been standardized (Marks et al. 2010). At our hospitals, all colorectal surgeons involved in this study had received formal training in TME, thus all patients in this group underwent surgical treatment involving adequate TME proctectomy. The inadequate LN sampling may suggest incomplete TME, which might result in adverse oncological outcome. However, some researchers were unable to identify any relationship between the reduced numbers of LNs examined and decreased patient survival (Rullier et al. 2008; Ha et al. 2010). Moreover, a study by the Cleveland Clinic not only confirmed the association between neoadjuvant therapy and decreased number of LNs retrieved from proctectomy specimens, but also suggested that the diminished number of LNs is actually an indicator of improved tumor response and consequently decreased local recurrence rates (Campos-Lobato et al. 2013).

LPLN metastasis is another reason for the local recurrence in our data. In the present study, the failure pattern of the three cases raises the question of whether LPLD should be performed in patients with suspected LPLN metastasis based on pre-CRT CT or MRI, and how patients who may benefit from LPLD can be identified. Studies from Japan have demonstrated that the incidence of LPLN metastasis is approximately 15% in patients with advanced low rectal cancer who underwent LPLD, and varies from 10.6 to 25.5% (Konishi et al. 2010). The major drawback is that the above mentioned results were obtained from patients who had not undergone preoperative CRT. In the Western countries, the standard treatment for patients with locally advanced rectal cancer is preoperative CRT followed by TME alone. LPLN metastasis is generally considered a metastatic disease that is not amenable to surgical cure. Moreover, some studies have revealed that preoperative radiotherapy followed by TME led to acceptable local control that was as good as that achieved by extended surgery, which included LPLD (Georgiou et al. 2009; Quadros et al. 2012). Consequently, it is widely accepted in the Western countries that routine LPLD is not necessary. However, several studies have revealed that selective LPLD might improve local control and survival in patients with LPLN metastasis in advanced low rectal cancer who have been treated with preoperative CRT. Akiyoshi and colleagues showed that the rate of pathological LPLN metastasis was 66% when they performed pre-CRT LPLD based on LPLN size and that preoperative CRT alone could not eradicate LPLN metastasis completely, thus LPLD might improve local control and survival of patients with LPLN metastasis in advanced low rectal cancer treated with preoperative CRT (Akiyoshi et al. 2014). Kim and colleagues showed that lateral pelvic recurrence is the most common pattern of local recurrence following neoadjuvant CRT plus TME alone and that patients with preoperative evidence of LPLN metastasis could benefit from LPLD even after neoadjuvant CRT (Kim et al. 2008). In our study, among the 195 patients who achieved pCR, CT scan or MRI detected lateral LN metastasis in ten patients with low rectal cancer, 3 (30%) of whom eventually developed lateral pelvic recurrence after follow-up. Although the evidence from our cohort is very limited, we agree with previous studies on the most common pattern of local recurrence, and that TME alone following preoperative CRT might be insufficient for controlling LPLN metastasis that is present before CRT. As suspicious metastatic LNs in the lateral pelvic area are a risk factor for local recurrence in patients who have undergone curative resection with preoperative concurrent CRT, and LPLD has been associated with increased morbidity such as blood loss, and urinary and sexual dysfunction, correct detection of LPLN is crucial. Previously, CT was the standard method for assessing the pre-treatment clinical nodal stage. Unfortunately, a meta-analysis (Bipat et al. 2004) reported only 55% sensitivity for LN involvement by CT staging. Consequently, more reliable imaging criteria for detecting LPLN metastasis are needed. Currently, newer techniques, e.g., MRI or positron emission tomography (PET) scanning are used to improve LN staging accuracy. Brown et al. (2003) found that high-spatial resolution MRI had a sensitivity of 85% [51/60 (95% confidence interval, CI: 74%, 92%)] and a specificity of 97% [216/221 (95% CI: 95%, 99%)] for LN (+) defined by the presence of mixed signal intensity or irregular nodal capsule border. Koh and colleagues (Koh et al. 2010) used ultra-small paramagnetic iron oxide (USPIO), a LN-specific biodegradable contrast agent with the potential to enable identification of nodal metastasis independently of LN size for MRI. They found that the USPIO enhancement pattern had higher diagnostic specificity, but the same sensitivity, as morphological findings in pathologically matched mesorectal LNs (specificity, 93 vs. 75%; sensitivity, 65 vs. 65%). It appears that PET/CT is less accurate than MRI for LN staging (Farwell et al. 2014), but has higher specificity than MRI (83–85% vs. 67%) in nodal staging (Kim et al. 2011; Cipe et al. 2013). Additionally, we believe that combined MRI and PET (fusion imaging) would increase the accuracy of nodal staging. Further studies are essential for identifying the most reliable imaging criteria for LPLN metastasis in patients treated with preoperative CRT.

The present study demonstrates that distant metastases are the major failure pattern of patients who achieve pCR after CRT, which is similar to the findings of previous reports (Table 2). Maas and colleagues reported a 5-year distant metastasis rate of 11.2% (Maas et al. 2010). Capirci and colleagues performed a long-term analysis of 566 patients who achieved pCR, reporting a distant metastasis rate of 8.9% after a 46-month follow-up (Capirci et al. 2008). The persistence of distant metastases suggests the inadequate systemic control of the CRT regimen. Thus, the high rate of distant failure may indicate that adjuvant chemotherapy is warranted in these patients. However, the role of postoperative CT following neoadjuvant CRT and radical surgery for patients with locally advanced rectal cancer is still not entirely clear, especially in patients with pCR. Some studies suggest that there is little or no benefit from chemotherapy (Maas et al. 2010; Capirci et al. 2008; Kiran et al. 2012). However, Collette and colleagues (Collette et al. 2007) demonstrated that the administration of adjuvant chemotherapy was associated with significantly improved overall survival. Their study randomly assigned patients to adjuvant chemotherapy or no adjuvant chemotherapy after (chemo) radiation and surgery for rectal cancer. Patients with ypT0–2 tumors, but not ypT3–4 tumors, benefited from adjuvant chemotherapy; the authors explained that tumors that respond well to preoperative FU-based chemoradiation would also be sensitive to adjuvant chemotherapy involving this drug. These contrasting findings indicate that more trials involving adjuvant chemotherapy following neoadjuvant CRT should be performed in future.

**Table 2:** Outcomes of patients with pCR at other hospitals

References	pCR	Distant metastasis (%)	Local recurrence (%)
Capirci et al. (2008)	536	8.9	0.9
De Campos-Lobato et al. (2011)	54	10	0
Smith et al. (2010)	100	8	1
Yeo et al. (2010)	304	7.9	2.6
Current study	195	7.7	1.5

*pCR* pathologic complete response

Consistent with previous reports, we also observed a unique pattern of distant recurrence, where there was a predominance of non-liver metastases. In our study, only one patient developed liver metastasis; extra-liver metastases involved seven lung metastases and two bone metastases. Different theories have been proposed to explain the pattern of metastases, although this process appears to be multifactorial (Ding et al. 2012). The “seed and soil” hypothesis states that, due to different organ-specific microenvironments, tumors may metastasize to specific organs independently from the vascular anatomy (Fidler 2003). However, some researchers suggest that tumors might metastasize to specific organs independently based on the differential level of thymidylate synthase (*TS*) mRNA, which causes differential sensitivity to 5-FU-based chemotherapy. Patients with higher *TS* mRNA levels easily develop pulmonary metastasis (Yamada et al. 2001). Such findings are also attributed to the nature of the venous drainage of the rectum (Watanabe et al. 2011). Intensive surveillance schemes and improved imaging technology may also contribute to the high rate of lung metastasis detected, which is consistent with that of previous studies (Kirke et al. 2007; Lee et al. 2007).

There are some potential limitations to this study. The first lies in the limited number of enrolled patients, given their good outcomes. The second potential limitation is that the number of LNs retrieved was much lower than that in previous reports on patients treated with neoadjuvant CRT. However, the inadequate LN sampling in our study may have been due to improved tumor response. The third potential limitation involves regular assessment of the circumferential resection margin (CRM) status. The CRM is considered one of the strongest predictors of surgical failure in rectal cancer (Kelly et al. 2011). We do not routinely assess the CRM status, but the pathologists at our hospitals routinely perform macroscopic evaluation of rectal cancer resection specimens. The quality of the mesorectum is classified as good (mesorectal), intermediate (intramesorectal), or poor (muscularis propria plane) (Quirke 2003). The mesorectum quality of the 18 patients included in our data was good, indicating smooth CRM on slicing.

In conclusion, our data demonstrate that distant metastases are the major failure pattern of patients who achieve pCR after CRT and that non-liver metastases are the predominant pattern of distant recurrence. Our results indicate the possible predominant pattern of distant recurrence and serve as a basis for managing LPLN metastasis in low rectal cancer. Patients with LPLN metastasis in advanced low rectal cancer treated with preoperative CRT would benefit from selective lateral LN excision.

## Abbreviations

5-FU: 5-Fluorouracil
AJCC: American Joint Committee on Cancer
APR: Abdominoperineal resection
AWD: Alive with disease
Cape: Capecitabine
chemo: Chemotherapy
CI: Confidence interval
CRM: Circumferential resection margin
CRT: Chemoradiotherapy
CT: Computed tomography
DOD: Died of disease
F: Female
FOLFOX6: 5-FU, leucovorin, oxaliplatin
LAR: Low anterior resection
LN: Lymph nodes
LPLD: Lateral pelvic lymph node dissection
LPLN: Lateral pelvic lymph node
M: Male
MRI: Magnetic resonance imaging
OS: Overall survival
Oxa: Oxaliplatin
pCR: Pathologic complete response
PET: Positron emission tomography
pN (n): Number of lymph nodes retrieved
RFS: Recurrence-free survival
RT: Radiotherapy
Tar: Targeted therapy
TME: Total mesorectal excision
USPIO: Ultra-small paramagnetic iron oxide
XELOX: Capecitabine and oxaliplatin
